# Lack of Association Between Intraoperative Hypotension and Postoperative Acute Kidney Injury in Patients Undergoing Pancreaticoduodenectomy: A Retrospective Cohort Study

**DOI:** 10.1155/ijne/5568151

**Published:** 2025-04-08

**Authors:** Yue Gu, Wenwen Zhang, Jing Zhou, Xiaoge Niu, Yanliang Wang, Limeng Wang, Lei Yan, Yang Xu, Fengmin Shao

**Affiliations:** ^1^Department of Nephrology, Henan Provincial Clinical Research Center for Kidney Disease, Henan Provincial Key Laboratory of Kidney Disease and Immunology, Henan Provincial People's Hospital, Zhengzhou University People's Hospital, Henan University People's Hospital, Zhengzhou, Henan, China; ^2^Peking University Clinical Research Institute, Peking University First Hospital, Beijing, China; ^3^Department of Medical Epidemiology and Biostatistics, Karolinska Institutet, Solna, Sweden

**Keywords:** acute kidney injury, intraoperative blood pressure management, intraoperative hypotension, pancreaticoduodenectomy, restricted cubic spline

## Abstract

**Background:** Acute kidney injury (AKI) is a common postoperative event. Previous research suggests that intraoperative hypotension (IOH) is associated with postoperative AKI. This connection, however, has not been studied in patients undergoing pancreaticoduodenectomy.

**Methods:** Based on a retrospective cohort study, we analyzed 844 adult patients who had pancreaticoduodenectomy between December 2016 and June 2020 in Henan Provincial People's Hospital. We graphically modeled the associations between the lowest intraoperative systolic and diastolic pressure and AKI using a restricted cubic spline with all covariates adjusted. The association between time under the above-specified systolic blood pressure (SPB) and diastolic blood pressure (DBP) thresholds and AKI, respectively, was investigated using logistic regression models. We further tested the robustness of our findings with a sensitivity analysis.

**Results:** AKI occurred in 98 (11.6%) of the 844 patients in this cohort. Blood pressure components below the thresholds of 100 mmHg for systolic and 60 mmHg for diastolic were visual change points associated with increasing odds of AKI. The median (IQR) time under SBP < 100 mmHg was 15.0 (0, 40) min and 65.0 (18.8, 105.4) min for DBP < 60 mmHg. Time spent under the threshold of SBP less than 100 mmHg and DBP less than 60 mmHg was not significantly associated with AKI.

**Conclusions:** We found no relationship between IOH and postoperative AKI after pancreaticoduodenectomy. More research is needed to investigate the complex aspects influencing intraoperative blood management in order to lessen the occurrence of AKI.

## 1. Introduction

Pancreatoduodenectomy is a classic surgical treatment for pancreatic, periampullary, and distal bile duct malignancies. The mortality rate of this procedure has reduced from > 20% to < 2% [1], although the incidence of complications remains as high as 29%–50% [2–4], with acute kidney damage (acute kidney injury [AKI]) accounting for 5.9%–20.4% [5–8]. AKI is a frequent postoperative occurrence linked to higher rates of death, longer hospital stays, higher medical expenses, and a higher chance of developing chronic kidney disease [7, 9, 10].

Several studies have highlighted male, age, obesity, and comorbidities such as hypertension, diabetes, coronary artery disease, peripheral vascular disease, and anemia as risk factors for postoperative AKI [11–13], the majority of which are difficult to control. In recent years, some researchers have shown that intraoperative hypotension (IOH), a manipulable factor, is strongly connected to the occurrence of postoperative AKI [14–17], while others had different findings [18, 19]. However, most of those studies suggesting a positive correlation between IOH and AKI characterized IOH as mean arterial pressure (MAP) below a certain threshold. Note that systolic and diastolic pressure also play an important role in hemodynamics, and therefore presumably influence the perfusion of the kidney in a different way. Moreover, these studies have been performed on patients receiving various types of surgeries, the heterogeneity of the study population may contribute to the inconsistency. As far as we are aware, not much research has been performed on individuals undergoing pancreaticoduodenectomy, one of the most challenging procedures in abdominal surgery. As a result, we performed a historical cohort analysis to assess the relationships between intraoperative systolic and diastolic pressure and AKI in patients undergoing pancreaticoduodenectomy.

## 2. Materials and Methods

### 2.1. Study Design and Population

This retrospective cohort study was carried out in Henan Provincial People's Hospital as a single center. The protocols adhered to were compliant with the 1975 Helsinki Declaration, as amended in 2000, and the ethical guidelines of the relevant committee on human experimentation (national and institutional) [20]. The study protocol was approved by the Ethics Committees of Henan Provincial People's Hospital (Ethics approval number: 2021-Lunshen-77). Informed consent was waived due to the retrospective nature of this study.

**Figure 1:**
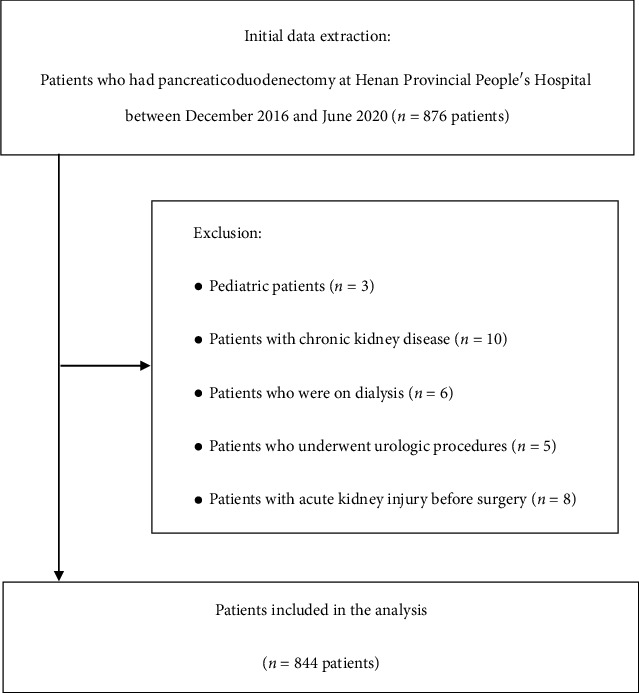
Flowchart of patients' exclusion process.

844 adult patients who underwent pancreaticoduodenectomy between December 2016 and June 2020 were included in the study, with preoperative and at least one postoperative serum creatinine measurement available within the first 7 postoperative days. Individuals who underwent urologic procedures (such as nephrectomy, renal transplantation, or relief of urinary obstruction), AKI prior to surgery, chronic kidney disease (defined as preoperative estimated glomerular filtration rate < 60 mL × min^−1^ × 1.73 m^−2^), or required dialysis were excluded from the study. Individuals undergoing urologic procedures were not included in the analysis due to the possibility that the changes in postoperative creatinine in these patients were more closely linked to the surgical intervention (Figure 1).

### 2.2. Exposure

We took the intraoperative blood pressure straight out of our electronic medical file. Clinicians cannot change it, but it can be detected as artifactual. Invasive systolic blood pressure (SBP) and diastolic blood pressure (DBP) were recorded every 5 min, and we also excluded readings we defined as an artifact using previously published criteria: SPBs ≥ 300 mmHg or ≤ 20 mmHg and DBPs ≤ 5 mmHg or ≥ 225 mmHg [16, 17, 21]. We interpolated pressure changes between measurements as linear. To assess the influence of IOH on AKI from different perspectives, we described IOH as (1) the lowest intraoperative systolic and diastolic of each patient, which represents the severity of IOH, and (2) the number of minutes under intraoperative systolic and diastolic pressure threshold for each patient, which represents the duration of IOH, defined as the total duration (*t*1 + *t*2 + *t*3…) of time spent under the specified threshold.

### 2.3. Outcome

The primary outcome was AKI, which is identified if any one of the following conditions was present, according to KDIGO 2012 [22]: an increase in SCr of ≥ 0.3 mg/dL (≥ 26.5 μmol/L) within 48 h, an increase in SCr of ≥ 1.5 times the baseline value within 7 days, or urine volume < 0.5 mL/kg/h for 6 h. The KDIGO urine output criteria were not applied in this investigation since the urine output of our postoperative group was unavailable.

### 2.4. Covariates

Study covariates were extracted from the electronic medical record database, including age, gender, comorbidities (e.g., hypertension, diabetes, coronary heart disease [CHD], and anemia), preoperative medication use (e.g., contrast agent, nonsteroidal anti-inflammatory drugs [NSAIDs], and diuretics), preoperative laboratory tests (albumin), and intraoperative factors (e.g., operation duration, red blood cell transfusion, urine output, colloid transfusion).

### 2.5. Statistical Analysis

Patients' characteristics are summarized in Table 1. Values are expressed as mean and SD for continuous variables with normal distribution, median (interquartile range) for non‐normal distribution variables, and percentage of total for categorical variables. The Mann–Whitney U-test or Pearson chi-square test was used to compare patients with and without postoperative AKI. The analysis of baseline data in this part is processed by SPSS Statistics Version 25.

First, we graphically modeled the associations between the lowest intraoperative systolic and diastolic pressure and AKI using restricted cubic spline (30 mmHg of SBP and 9 mmHg of DBP served as the reference level, respectively) with all covariates adjusted. From the graphics, thresholds of SBP and DBP for calculating the duration of IOH were chosen visually.

Then, we performed logistic regression for models to examine the relationship between time under the above-specified SBP and DBP thresholds and AKI, respectively. All patients were divided into two groups according to the median value of the time under specified SBP and DBP thresholds. Three separate models were constructed for these analyses: Model 1 was not adjusted for covariates; Model 2 was adjusted for age and gender; and Model 3 was additionally adjusted for hypertension, diabetes, CHD, concurrent medication before surgery, contrast agent, NSAIDs, diuretics, albumin, anemia, operation duration, intraoperative red blood cell transfusion, intraoperative urine output, and intraoperative colloids transfusion.

There were missing values for albumin, anemia, and operation duration, with missing rates of 0.1%, 0.1%, and 0.4%, respectively. We used chained equations by classification and regression trees (CARTs) to impute 5 complete datasets for each outcome separately. The predictors included both observed patient characteristics and the event indicator for the outcome. Adjusted analyses were performed on the five imputed datasets, and then the results were combined.

Subgroup analyses were performed by categories of age and sex. We also conducted a sensitivity analysis to test the robustness of our results. Instead of choosing the thresholds of SBP and DBP for the calculation of the IOH duration visually, we chose thresholds by the threshold regression model [23]. Data were analyzed using R Version 3.6.0 (R Foundation for Statistical Computing, Vienna, Austria). All tests were two-sided, and was considered statistically significant.

## 3. Results

### 3.1. Baseline Characteristics

Baseline characteristics of the study patients are presented in Table 1. The median (IQR) age of patients was 60.0 (51.0, 67.0) years, and 58.3% were males. The most common complication of patients was anemia (31.1%), followed by hypertension (26.4%) and diabetes (16.7%). 81.8% of patients used contrast agents before surgery, 9.1% used NSAIDs, and 17.1% used diuretics. The median (IQR) operation duration was 370.0 (315.0, 455.0) minutes.

### 3.2. Relationships Between IOH and AKI

**Figure 2:**
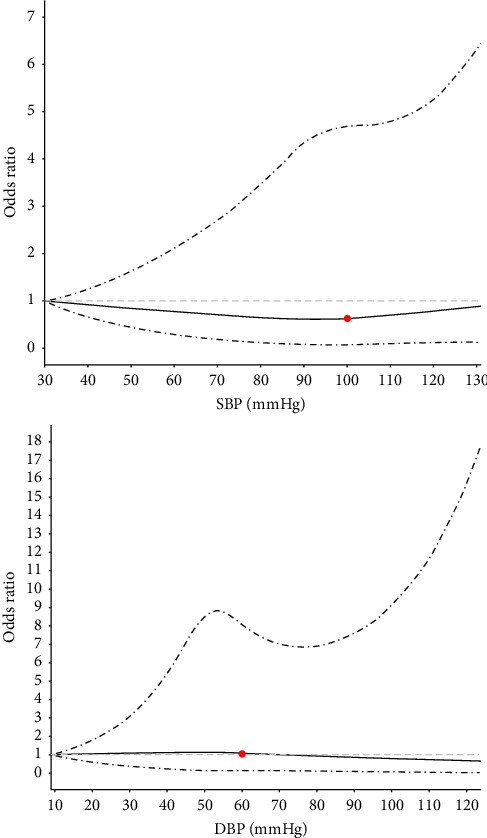
Dose-response relations between lowest blood pressure values and AKI after pancreatoduodenectomy. DBP: diastolic blood pressure; SBP: systolic blood pressure.

Restricted cubic spline smoothing plots for the lowest blood pressure components for each patient are shown in Figure 2. There was an inverse, U-shaped association between the lowest SBP and AKI, whereas the trend for the lowest DBP was not observed. Blood pressure components below the thresholds of 100 mmHg for systolic and 60 mmHg for diastolic were visual change points associated with increasing odds of AKI. The distribution of SBP and DBP is presented in Supporting Information (Supporting Figure 1).

Odds ratios were estimated using a restricted cubic spline function by comparing to a reference value of 30 mmHg for SBP and 9 mmHg for DBP. The solid line represents a point estimate and the dashed lines represent 95% confidence intervals.

### 3.3. Relationships Between Time Under Blood Pressure Thresholds and AKI

The median (IQR) time under SBP < 100 mmHg and DBP < 60 mmHg was 15.0 (0, 40) min and 65.0 (18.8, 105.4) min, respectively. Time spent under the threshold of SBP less than 100 mmHg and DBP less than 60 mmHg was not significantly associated with AKI (Table 2). Compared to patients with intraoperative SBP < 100 mmHg for less than 15 min, the estimated OR of AKI was 0.81 (95% CI: 0.51–1.27, *p*=0.360) for patients with intraoperative SBP < 100 mmHg for over 15 min in the fully adjusted model. For DBP, the estimated OR was 1.28 (95% CI: 0.81–2.01, *p*=0.293).

### 3.4. Subgroup and Sensitivity Analyses

Analyses stratified by age and gender revealed similar effects with main analyses (Table S1). We further conducted a sensitivity analysis using the new threshold obtained by the threshold regression models. From the threshold regression models, the estimated change point was 107 mmHg for SBP and 61 mmHg for DBP. We repeated the association analysis of intraoperative time under the new SBP/DBP thresholds with AKI and obtained similar results (Table S2).

## 4. Discussion/Conclusion

We analyzed all patients admitted to Henan Province People's Hospital for pancreaticoduodenectomy over a 4 year period to explore the relationship between IOH and AKI. By using the restricted cubic spline smoothing plots, we reported a U-shaped association between the lowest SBP and AKI, and, the thresholds of 100 mmHg for systolic and 60 mmHg for diastolic were visual change points associated with increasing odds of AKI. However, no relationship between the levels of IOH and AKI has been found in this study.

As a common and series complication of hospitalizations, AKI has a high in-hospital mortality rate range of 8.8%–23.8% [24, 25]. The incidence of AKI varies significantly depending on the area and type of surgery [26]. In this study, we reported a 11.6% pooled incidence of postoperative AKI following pancreaticoduodenectomy. In a large Japanese study of 84,036 patients, the authors compared robotic-assisted versus open pancreaticoduodenectomy and found the AKI incidence was 7.0% and 6.5% in the robotic-assisted group and open group, respectively [5]. Mahmooth et al. [6] observed a significant rate of surgical AKI, with 19.7% of 395 patients suffering from postoperative AKI. Their study [6] estimated an incidence of AKI that was almost twice as high as ours, and this discrepancy may be due to the surgical approach and perioperative management. Park et al. found an AKI incidence of 5.3% after pancreaticoduodenectomy in a group of 809 patients [8]. However, the investigators [8] only enrolled patients who underwent pylorus-preserving pancreaticoduodenectomy but not conventional pancreaticoduodenectomy, which may understate the prevalence of AKI.

AKI progression is difficult and influenced by a variety of factors, including preoperative physiopathological conditions, anesthesia, type of surgery, and postoperative management [27]. IOH, a frequent side effect of general anesthesia, can raise the risk of AKI by interfering with organ perfusion [28]. However, previous studies about the relationship between IOH and AKI are controversial. In our study, we reported there was no relationship between the IOH and AKI. Several other studies [18, 19, 29, 30] that compared groups with and without AKI did not find any difference in the degree of IOH, which is consistent with our findings. Kluger et al. [18] assessed the relationship among AKI, mortality, length of stay, and IOH (defined as a MAP ≤ 55, 60, 65, 70, and 75 mmHg) in 1063 elderly patients undergoing unilateral hip fracture surgery and discovered that there was no correlation between AKI and the length of hypotension or IOH. In a study examining intraoperative blood pressure in 2431 unilateral total hip arthroplasty patients, no association was identified between increased odds of postoperative AKI and a longer duration of hypotension [29]. Comparably, the effect of IOH was evaluated in a cohort study of patients undergoing nephrectomy, and no relationship between AKI and longer duration of IOH was identified [30].

In addition, there is some evidence to suggest that IOH and unfavorable renal events may be related [21, 31, 32]. Using relative SBP thresholds, Hallqvist et al. discovered that an intraoperative SBP drop of more than 50% was related to a more than 2-fold increase in the risk of AKI [31]. Another retrospective analysis with 248 patients discovered that MAP < 55 mmHg and SBP < 80 mmHg were significant risk variables for postoperative AKI [32]. These investigations, however, did not look at the relationship between hypotension duration and AKI. A recent study demonstrates that even brief IOH increases the risk of AKI [17]. Finally, Ahuja et al. showed in a cohort of 23,140 noncardiac surgery patients that the lowest SBP and duration at the lowest SBP could progressively increase the risks of myocardial or kidney injury [21]. Although this study investigated the role of all components of arterial pressure (including systolic, diastolic, and mean pressure) in AKI, the included population [21] was different compared to ours. Our study included only pancreaticoduodenectomy patients to create a homogeneous population.

Among the previous studies with a controversial conclusion about the relationship between IOH and AKI, the standards for the IOH were different. Most of the studies used MAP less than 55–75 mmHg [18, 19, 29, 30, 32]. Kluger et al. [18] defined IOH as a MAP ≤ 55, 60, 65, 70, and 75 mmHg in a cohort of 1063 elderly patients undergoing unilateral hip fracture surgery. Braüner Christensen et al.'s [19] collected MAP at any point during anesthesia was ≤ 55 mmHg. In a study of 2431 individuals undergoing unilateral total hip arthroplasty, hypotension was defined as MAP less than 60 mmHg [29]. In a cohort study of nephrectomy patients, MAP was < 55 mmHg [30]. Similarly, in another retrospective study including 248 participants, MAP was also described as < 55 mmHg [32]. The different standards can potentially result in misleading results [33]. The different types of surgery and medical conditions also affect the risk of AKI [33]. Hence, in our study, we used the restricted cubic spline smoothing plots to define the blood pressure threshold, which was 100 mmHg for systolic and 60 mmHg for diastolic, thus providing a relatively more accurate estimate for patients with pancreatoduodenectomy under the same medical conditions.

Based on the threshold, we further analyzed the associations between AKI and blood pressure. We found no associations between blood pressure and AKI, or between AKI and time under the blood pressure threshold. A sensitivity analysis with IOH yielded similar results, supporting the lack of a significant association between IOH and AKI.

The anesthesia and blood pressure management during the operation have a significant impact on the occurrence of AKI [34]. The renal function was much more strongly related to the severity of IOH than the duration of hypotension [15]. The maintenance of hemodynamic stability, oxygen-carrying capacity, and perfusion of vital organs is critical to reducing the incidence of AKI [35]. Pancreaticoduodenectomy is a complicated and time-consuming (around 6 h) procedure in which anesthesiologists play a critical role. In our study, the anesthesiologists controlled the intraoperative blood pressure at a range between 20% above and below the base blood pressure. This personalized intraoperative blood pressure management might contribute to the stable intraoperative hemodynamic, leading to the low incidence of severe IOH which affects the renal function [36].

Unlike previous studies which included a diverse population, we performed a comprehensive evaluation of IOH in a specific population undergoing pancreaticoduodenectomy. In addition, we also performed the sensitivity analysis to choose SBP/DBP thresholds statistically. Results from sensitivity analysis were consistent with the primary analysis, which double-confirmed the robustness of our findings. Despite these strengths, our study also has limitations. Our study was a single-center study including a relatively small sample size, thus the results may not be generalizable to other populations who had surgery with different perioperative hemodynamic and anesthetic management. In addition, arterial blood pressure measurements in our study were taken every 5 min, which may have been less accurate than other studies' blood pressure readings that were taken every 0.5–2 min [16, 18, 29].

To lower the occurrence of AKI, a thorough understanding of the pathophysiology of hematologic derangements as well as a multimodal patient blood management strategy are required. In our study, we defined a threshold to study the relationship between IOH and AKI in a specific population that received pancreatoduodenectomy, which provided a potential reference standard for further study. Based on this threshold, there was no association between IOH and AKI after pancreaticoduodenectomy was reported. This might be explained by the personalized blood management during the operation. Further studies are needed to explore the complicated factors affecting intraoperative blood management to reduce the incidence of AKI.

## Figures and Tables

**Table 1:** Baseline characteristics of the study patients.

Characteristic	All participants (*n* = 844)	AKI group (*n* = 98)	Non-AKI group (*n* = 746)	*p*
Age, years	60.0 (51.0, 67.0)	61.0 (52.0, 69.0)	60.0 (51.0, 67.0)	0.119
Male (%)	492 (58.3)	63 (62.3)	429 (57.5)	0.201
Hypertension (%)	223 (26.4)	30 (30.6)	193 (25.9)	0.317
Diabetes (%)	141 (16.7)	20 (20.4)	121 (16.2)	0.296
CHD (%)	58 (6.9)	8 (8.2)	50 (6.7)	0.591

*Concurrent medication before surgery*				
Contrast agent (%)	690 (81.8)	83 (84.7)	607 (81.4)	0.423
NSAIDs (%)	144 (17.1)	15 (15.3)	129 (17.3)	0.623
Diuretics (%)	77 (9.1)	15 (15.3)	62 (8.3)	0.024
Albumin (g/L)	37.6 (34.0, 40.8)	37.2 (33.8, 40.9)	37.7 (34.1, 40.8)	0.694
Anemia (%)	262 (31.0)	33 (33.7)	229 (30.7)	0.555
Operation duration (min)	370.0 (315.0, 455.0)	370.0 (311.5, 520.0)	370.0 (315.0, 450.0)	0.286
Intraoperative red blood cell transfusions (U)	0.0 (0.0, 4.0)	0.0 (0.0, 4.0)	0.0 (0.0, 4.0)	0.927
Intraoperative urine output (mL)	775.0 (500.0, 1000.0)	700.0 (450.0, 1000.0)	800.0 (500.0, 1000.0)	0.219
Intraoperative colloid transfusion (mL)	500.0 (500.0, 1000.0)	650.0 (500.0, 1000.0)	500.0 (500.0, 1000.0)	0.173

*Note:* Continuous variables are presented as median (IQR) and categorical variables as numbers (percentages).

Abbreviations: CHD, coronary heart disease; NSAIDs, nonsteroidal anti-inflammatory drugs.

**Table 2:** Associations between AKI and time under blood pressure threshold.

Model	Time under SBP < 100 mmHg		Time under DBP < 60 mmHg	
	OR	*p*	OR	*p*
Model 1	0.86 (0.56–1.32)	0.500	1.42 (0.93–2.18)	0.105
Model 2	0.90 (0.58–1.38)	0.625	1.31 (0.84–2.02)	0.232
Model 3	0.81 (0.51–1.27)	0.360	1.28 (0.81–2.01)	0.293

*Note:* Logistic regression models were used to calculate the ORs. Model 1 with no adjustment. Model 2 was adjusted for age and gender. Model 3 was adjusted for age, gender, hypertension, diabetes, CHD, contrast agent, NSAIDs, diuretics, albumin, anemia, operation duration, intraoperative red blood cell transfusion, intraoperative urine output, and intraoperative colloid transfusion.

Abbreviations: AKI, acute kidney injury; CHD, coronary heart disease; DBP, diastolic blood pressure; NSAIDs, nonsteroidal anti-inflammatory drugs; OR, odds ratio; SBP, systolic blood pressure.

## Data Availability

The data used to support the findings of this study are available from the corresponding author upon request.

## Supporting Information

Supporting Figure 1: The distribution of SBP and DBP in our study.

Supporting Table 1: Associations between AKI and time under blood pressure threshold across different subgroups.

Supporting Table 2: Associations between AKI and time under blood pressure threshold.
