# Supplementary figures and images for: Lack of Association Between Intraoperative Hypotension and Postoperative Acute Kidney Injury in Patients Undergoing Pancreaticoduodenectomy: A Retrospective Cohort Study
Source: Int J Nephrol. 2025 Apr 8;2025:5568151. doi: 10.1155/ijne/5568151 (PMC11999749; doi:10.1155/ijne/5568151)

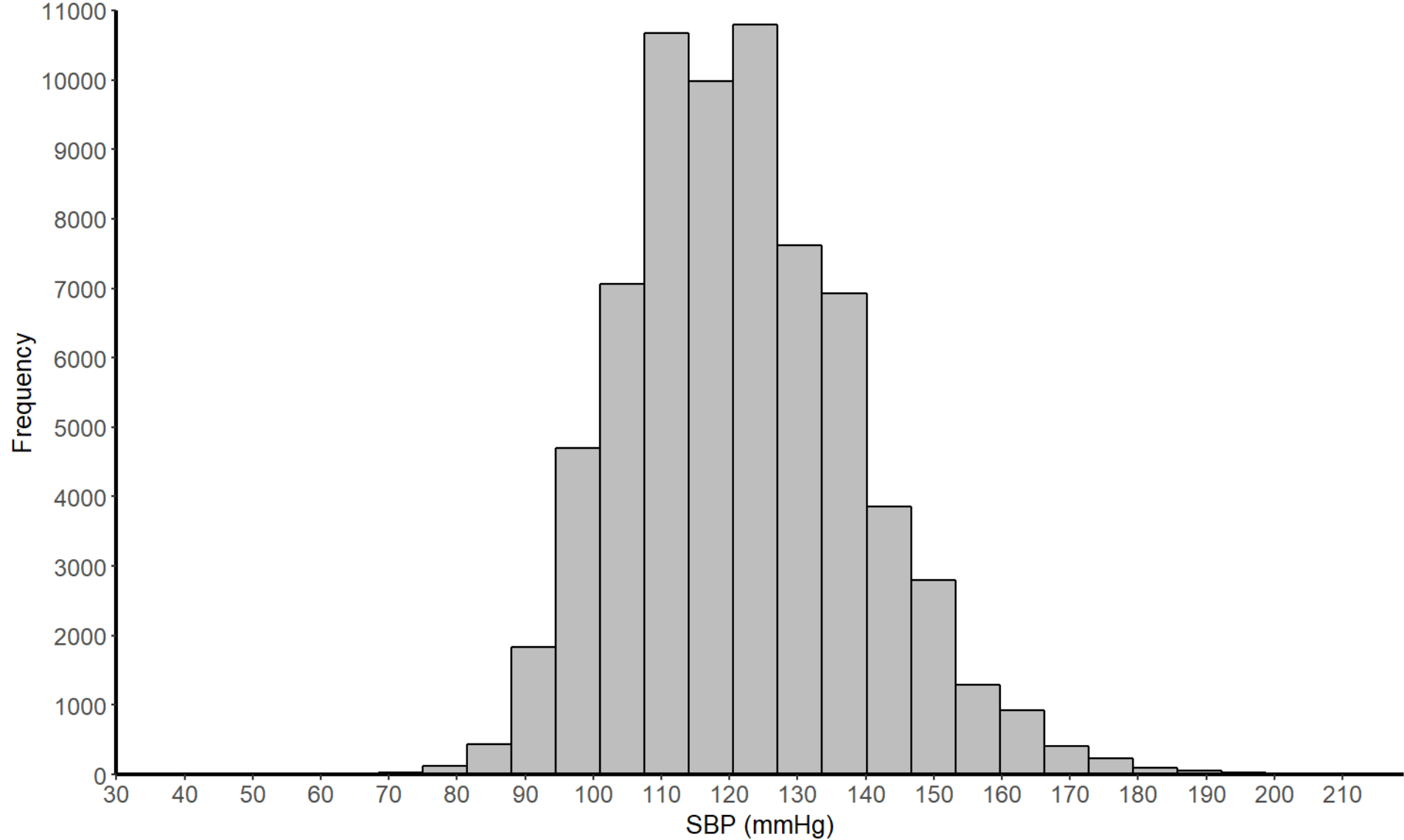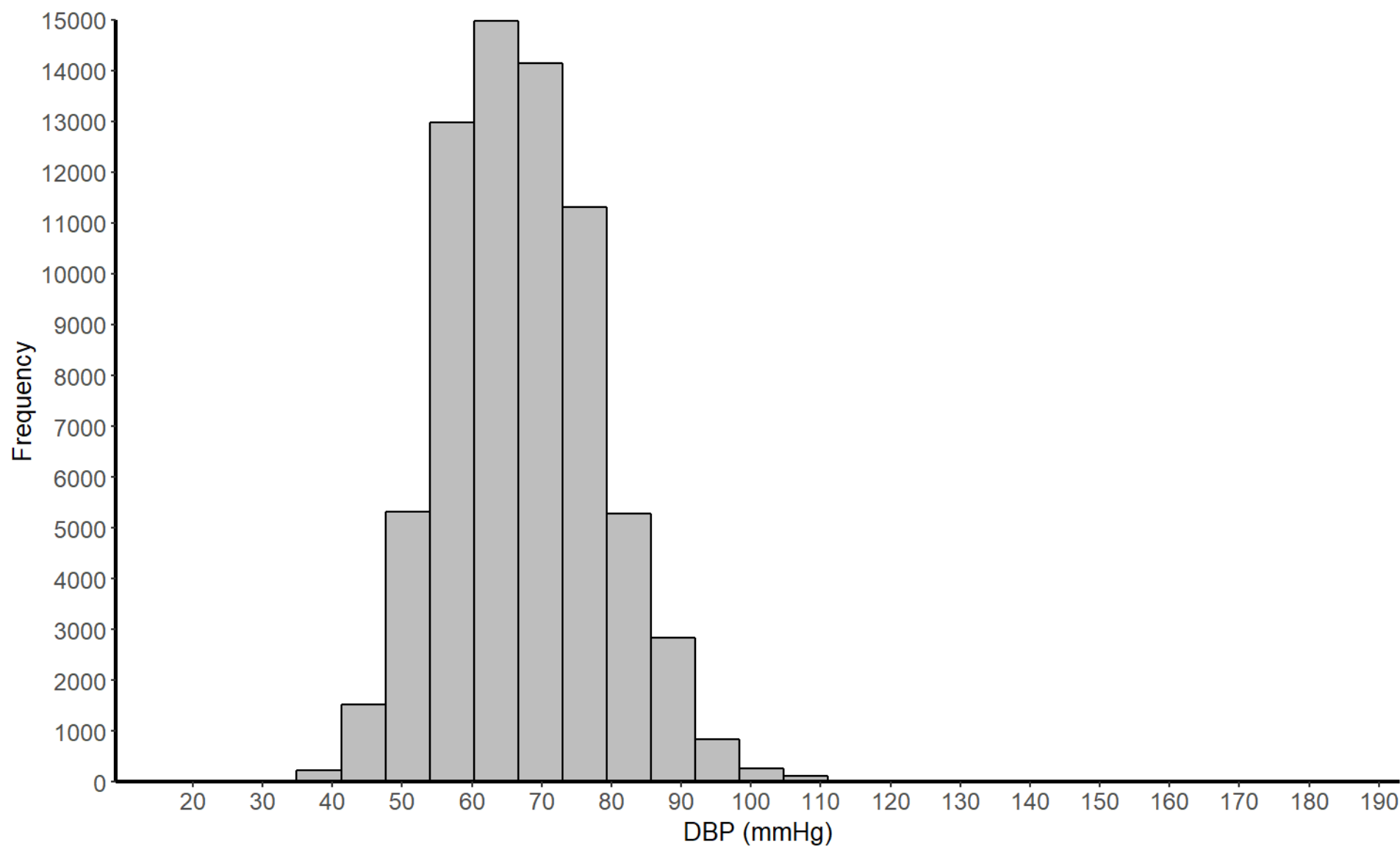

Supplement: Supporting Information — Additional supporting information can be found online in the Supporting Information section. [file 5568151.f1.zip › Supplementary Figure 1 (1).pdf]
