# Supplementary material for: Lack of Association Between Intraoperative Hypotension and Postoperative Acute Kidney Injury in Patients Undergoing Pancreaticoduodenectomy: A Retrospective Cohort Study
Source: Int J Nephrol. 2025 Apr 8;2025:5568151. doi: 10.1155/ijne/5568151 (PMC11999749; doi:10.1155/ijne/5568151)
Supplement: Supporting Information — Additional supporting information can be found online in the Supporting Information section. [file 5568151.f1.zip › Supplementary Information.doc]

***Supplementary Material***

**Contents:**

**Supplementary Figure 1：**The distribution of SBP and DBP in our study.

**Supplementary Table 1：**Associations between AKI and time under blood pressure threshold across different subgroups

**Supplementary Table 2：**Associations between AKI and time under blood pressure threshold

**Supplementary Figure 1.** The distribution of SBP and DBP in our study. SBP, systolic blood pressures; DBP, diastolic blood pressures.

**Supplementary Table 1.** Associations between AKI and time under blood pressure threshold across different subgroups.

| Subgroup | Time under SBP < 100 mmHg | | Time under DBP < 60 mmHg | |
| --- | --- | --- | --- | --- |
|  | OR | *P* | OR | *P* |
| Age |  |  |  |  |
| <60 years | 1.08 (0.55-2.14) | 0.815 | 1.35 (0.68-2.69) | 0.385 |
| ≥60 years | 0.68 (0.36-1.30) | 0.244 | 1.50 (0.80-2.81) | 0.212 |
| Sex |  |  |  |  |
| Female | 0.47 (0.21-1.05) | 0.067 | 0.82 (0.38-1.76) | 0.607 |
| Male | 1.09 (0.62-1.92) | 0.763 | 1.71 (0.95-3.06) | 0.071 |

Logistic regression models were used to calculate the ORs.

Model 1 with no adjustment.

Model 2 was adjusted for age and gender.

Model 3 was adjusted for age, gender, hypertension, diabetes, CHD, contrast agent, NSAIDs, diuretics, albumin, anemia, operation duration, intraoperative red blood cell transfusion, intraoperative urine output, and intraoperative colloids transfusion.

AKI, acute kidney injury; SBP, systolic blood pressures; DBP, diastolic blood pressures; OR, odds ratio; CHD, coronary heart disease; NSAIDs, non-steroidal anti-inflammatory drugs.

**Supplementary Table 2.** Associations between AKI and time under blood pressure threshold.

| Model | Time under SBP < 107 mmHg | | Time under DBP < 61 mmHg | |
| --- | --- | --- | --- | --- |
|  | OR | *P* | OR | *P* |
| Model 1 | 0.96 (0.63-1.47) | 0.857 | 1.45 (0.95-2.22) | 0.087 |
| Model 2 | 1.01 (0.66-1.54) | 0.975 | 1.34 (0.86-2.07) | 0.194 |
| Model 3 | 0.90 (0.57-1.43) | 0.667 | 1.31 (0.83-2.07) | 0.237 |

Logistic regression models were used to calculate the ORs.

Model 1 with no adjustment.

Model 2 was adjusted for age and gender.

Model 3 was adjusted for age, gender, hypertension, diabetes, CHD, contrast agent, NSAIDs, diuretics, albumin, anemia, operation duration, intraoperative red blood cell transfusion, intraoperative urine output, and intraoperative colloids transfusion.

AKI, acute kidney injury; SBP, systolic blood pressures; DBP, diastolic blood pressures; OR, odds ratio; CHD, coronary heart disease; NSAIDs, non-steroidal anti-inflammatory drugs.
